# Striving for Improved Prognostication in Adults With Repaired Aortic Coarctation: Utility of the H_2_FPEF Score∗

**DOI:** 10.1016/j.jacadv.2022.100147

**Published:** 2022-11-30

**Authors:** Matthias Greutmann

**Affiliations:** Department of Cardiology, University Heart Center, University of Zurich, Zurich, Switzerland

**Keywords:** aortic coarctation, H_2_FPEF score, heart failure

**Figure:**
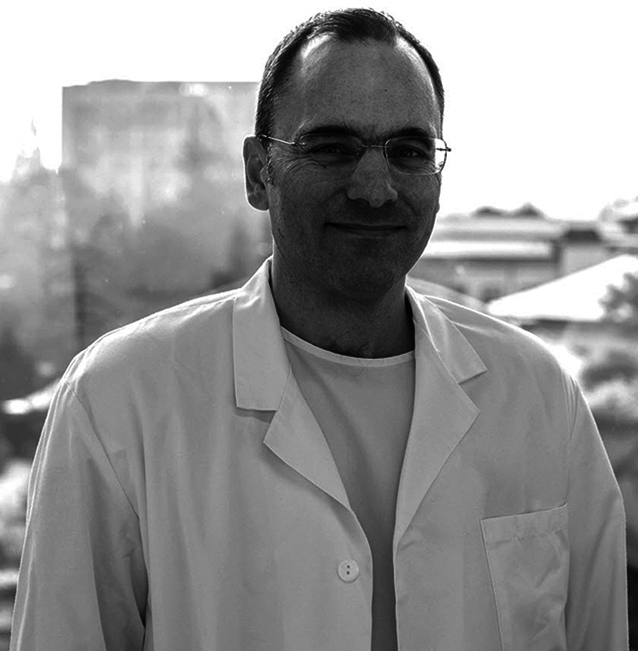

## Adults with repaired aortic coarctation

Aortic coarctation is a common congenital heart defect, accounting for about 10% of patients followed up at specialized centers.^1^ While aortic coarctation, on first glance, may appear to be a “simple” lesion, outcome studies have shown that it bears a substantially increased mortality risk, with heart failure being the predominant cause of attrition in affected young and middle-aged adults, accounting for 31% of deaths in one study.^2^

**Figure 1:**
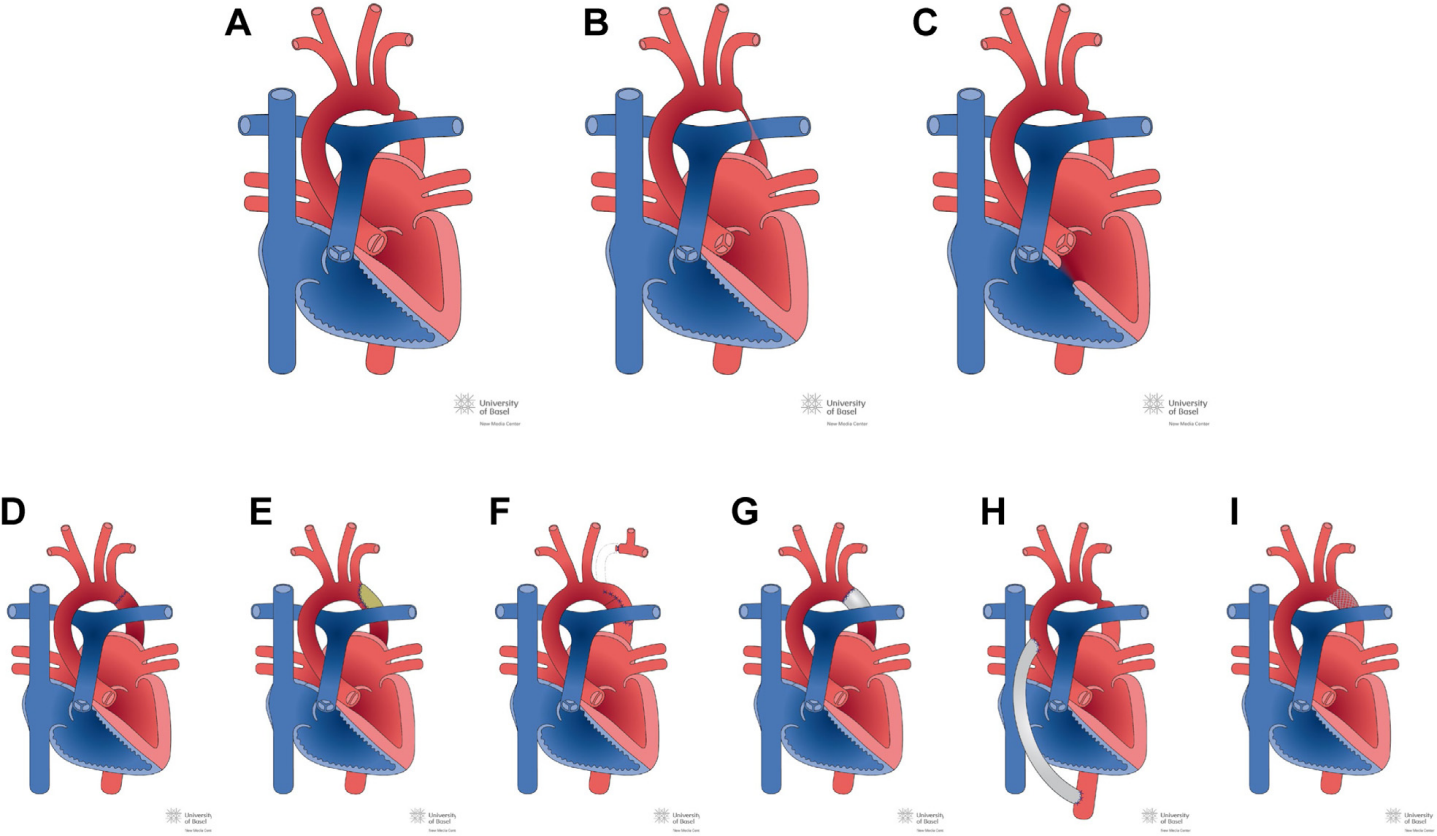
**Anatomic Variants of Aortic Coarctation and Different Repair Procedures** Illustration taken from http://www.chd-diagrams.com. **(A to C)** Variants of aortic coarctation and associated lesions: **(A)** focal type of aortic coarctation with concomitant bicuspid aortic valve; **(B)** aortic coarctation, tubular type; **(C)** aortic coarctation associated with muscular septal defects. **(D-I)** Types of repair operation: **(D)** repair by resection and end-to-end anastomosis; **(E)** repair by patch aortoplasty; **(F)** repair by subclavian flap aortoplasty; **(G)** repair by interposition graft; **(H)** repair by extra-anatomic ascendens-descendens graft; **(I)** repair by endovascular stent placement.

Aortic coarctation encompasses a spectrum of anatomical variants with a variety of associated lesions, most commonly bicuspid aortic valves, present in about two-thirds of all patients (Figures 1A to 1C). Furthermore, numerous surgical and interventional repair techniques have evolved over previous decades. These are tailored to the patient’s individual defect anatomy but also to evolving knowledge about long-term complications and progress in medical technology (Figures 1D to 1I). All these different repair techniques harbor the potential for long-term complications, generic to the defect or being specific to the type of individual repair technique. Thus, for affected patients, regular follow-up at specialized centers is mandatory.

## Heart failure in adult congenital heart disease: a major challenge ahead of us

Heart failure is the leading cause of death among adult survivors with congenital heart defects.^2^ With the evolution of novel adult patient cohorts, its overall prevalence and clinical importance will likely increase over upcoming decades.^3^ While the cohort of adults with end-stage heart failure is expected to increase substantially in the near future, only a minority of these patients will have access to timely heart transplantation.^4^ Identifying patients at risk, in order to optimize their therapy, with the aim of delaying the need for heart transplantation is therefore of paramount importance.

While there is no doubt that risk-stratification has to occur on a lesion-specific basis, even within a single disease entity, such as aortic coarctation, the spectrum of underlying anatomical variants and various types of repair techniques hamper the generalizability of risk scores based on these variables. More generic risk scores, such as the H_2_FPEF score, take into account demographic and hemodynamic characteristics and may overcome some of the variabilities among individual patients.

In their study in this issue of *JACC: Advances*, Egbe et al^5^ impressively confirms the high risk of serious cardiovascular complications among adults with repaired aortic coarctation. Within their cohort, over a median follow-up of 8 years, cardiovascular complications occurred in 14% of all patients, with heart failure being the predominant clinical problem occurring in 9% of the entire study cohort. They also found that a higher H_2_FPEF score was independently associated with an increased risk of cardiovascular events, particularly in patients with isolated aortic coarctation. Furthermore, in patients with follow-up of more than 5 years, the authors were able to demonstrate that an increase in the H_2_FPEF score during follow-up was independently associated with an increased risk of cardiovascular events. As expected, the H_2_FPEF score correlated with N-terminal pro-B-type natriuretic peptide levels (available in about 40% of patients) but interestingly not with left ventricular ejection fraction, nor with systolic pressure gradients across the coarctation site or with arm-leg pressure gradients.

## Outlook and future needs

Retrospective single-center studies are important in the field of adult congenital heart disease. Such studies allow the analysis of data with high granularity from single institutions with standardized follow-up protocols. However, such retrospective studies typically can generate hypotheses only, as results are subject to numerous biases by nature of data and analysis. Thus, it is important that hypotheses generated by retrospective studies be validated in prospective studies or registries, ideally in a multicenter setting.

There is no doubt that the validity of the H_2_FPEF score merits further assessments in such a prospective study. This will allow to identify whether the H_2_FPEF score and its changes over time will really prove to be an independent predictor of outcomes, independent, for example, from N-terminal pro-B-type natriuretic peptide levels that may be easier to obtain. Furthermore, longitudinal studies may allow to assess whether interventions such as aggressive treatment of systemic arterial hypertension or interventional treatment of residual coarctation may have an impact on H_2_FPEF scores and cardiovascular complications.

## Funding support and author disclosures

The author has reported that he has no relationships relevant to the contents of this article to disclose.
